# Supplementary material for: Microbiota of Urine, Glans and Prostate Biopsies in Patients with Prostate Cancer Reveals a Dysbiosis in the Genitourinary System
Source: Cancers (Basel). 2023 Feb 23;15(5):1423. doi: 10.3390/cancers15051423 (PMC10000660; doi:10.3390/cancers15051423)
Supplement: Supplementary file 1 [file cancers-15-01423-s001.zip › Supplementary Figures.docx]

Microbiota of Urine, Glans and Prostate Biopsies in Patients with Prostate Cancer Reveals a Dysbiosis in the Genitourinary System

Micael F. M. Gonçalves ^1^†*, Teresa Pina-Vaz ^2,3^†, Ângela Rita Fernandes ^1^, Isabel M. Miranda ^3,4^, Carlos Martins Silva ^2,3^, Acácio Gonçalves Rodrigues ^1,5^, Carmen Lisboa ^1,5,6^

**Supplementary Figures:**

**
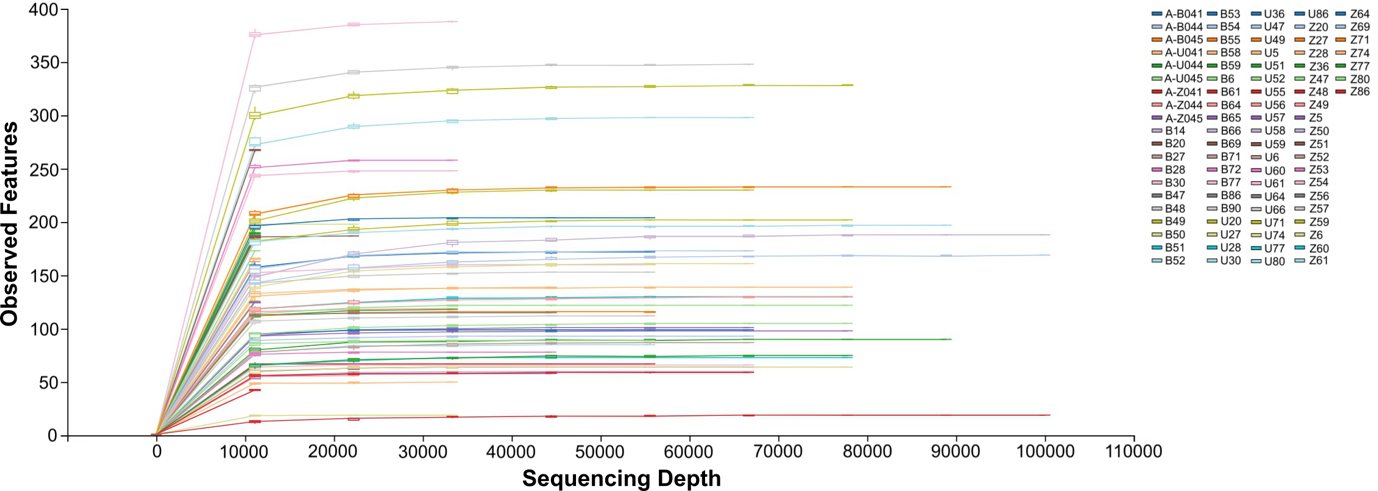
**

**Figure S1**: Rarefaction curves of the samples


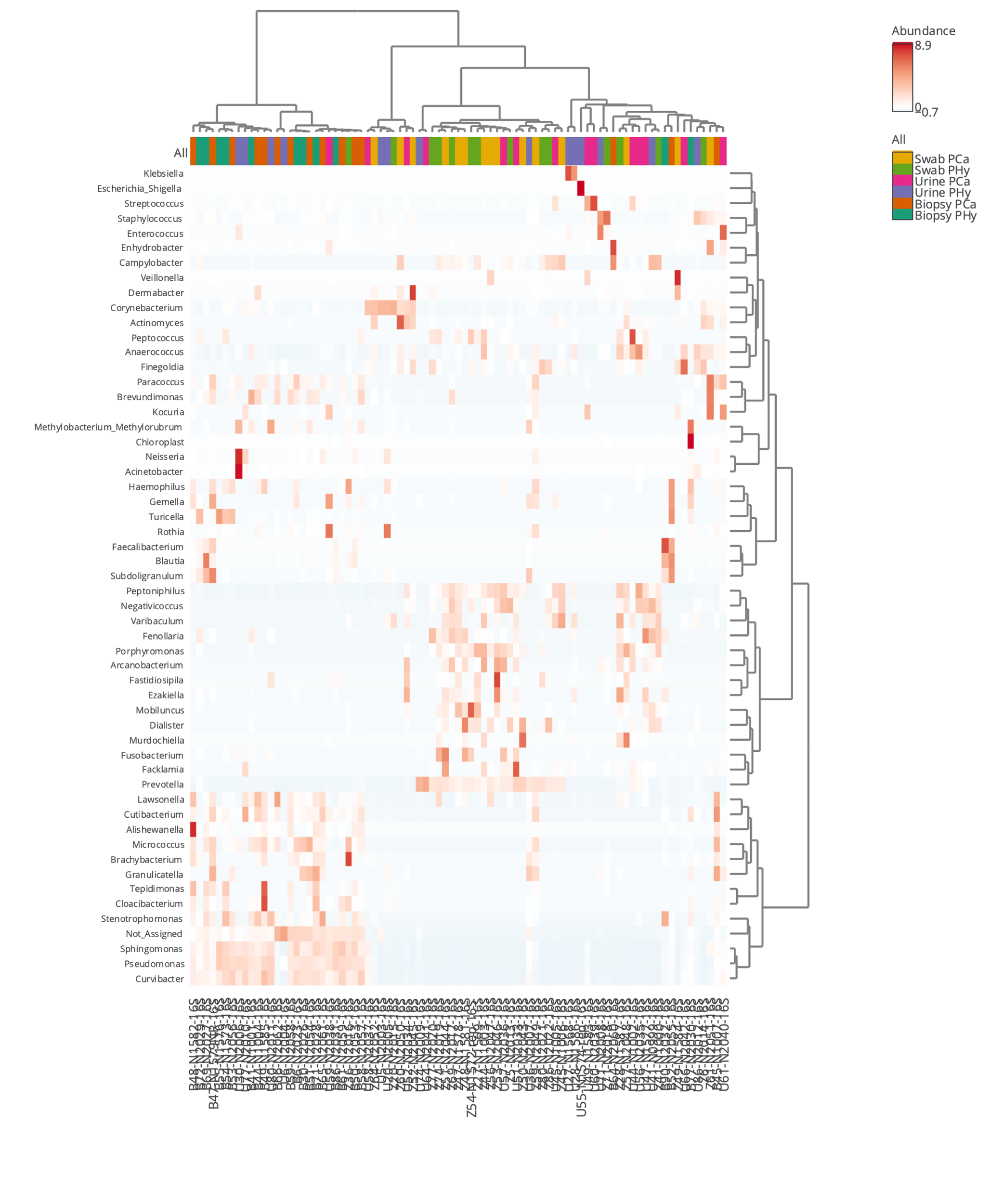
**Figure S2**: Heatmap for all genera represented in the urine, glans, and prostate biopsies samples for both PCa and non-PCa patients
